# Greater central airway luminal area in people with COVID-19: a case–control series

**DOI:** 10.1038/s41598-022-22005-6

**Published:** 2022-10-26

**Authors:** Jeffrey L. Jeltema, Ellen K. Gorman, Erik A. Ovrom, Juan G. Ripoll, Paolo B. Dominelli, Michael J. Joyner, Brian T. Welch, Jonathon W. Senefeld, Chad C. Wiggins

**Affiliations:** 1 Alix School of Medicine, Mayo Clinic, Rochester, MN USA; 2 Department of Anesthesiology and Perioperative Medicine, Mayo Clinic, 200 First Street SW, Rochester, MN 55905 USA; 3 Department of Kinesiology, University of Waterloo, Waterloo, ON Canada; 4 Department of Radiology, Mayo Clinic, Rochester, MN USA

**Keywords:** Infectious diseases, Respiration

## Abstract

Respiratory epithelium in the conducting airways of the human body is one of the primary targets of SARS-CoV-2 infection, however, there is a paucity of studies describing the association between COVID-19 and physical characteristics of the conducting airways. To better understand the pathophysiology of COVID-19 on the size of larger conducting airways, we determined the luminal area of the central airways in patients with a history of COVID-19 compared to a height-matched cohort of controls using a case–control study design. Using three-dimensional reconstruction from low-dose high-resolution computed tomography, we retrospectively assessed airway luminal cross-sectional area in 114 patients with COVID-19 (66 females, 48 males) and 114 healthy, sex- and height-matched controls (66 females, 48 males). People with a history of smoking, cardiopulmonary disease, or a body mass index greater than 40 kg·m^−2^ were excluded. Luminal areas of seven conducting airways were analyzed, including trachea, left and right main bronchus, intermediate bronchus, left and right upper lobe, and left lower lobe. For the central conducting airways, luminal area was ~ 15% greater patients with COVID-19 compared to matched controls (*p* < 0.05). Among patients with COVID-19, there were generally no differences in the luminal areas of the conducting airways between hospitalized patients compared to patients who did not require COVID-19-related hospitalization. Our findings suggest that males and females with COVID-19 have pathologically larger conducting airway luminal areas than healthy, sex- and height-matched controls.

## Introduction

Severe acute respiratory syndrome coronavirus 2 (SARS-CoV-2), the causative agent of novel coronavirus disease 2019 (COVID-19), is primarily transmitted through respiratory droplets, and attaches to ciliated epithelium in the conducting airways of the respiratory tree^1–3^. Cells of the conducting airways and gas exchange surfaces of the lungs share a common receptor for SARS-CoV-2, and much of the COVID-related symptomatology manifests in the conducting airways and lungs^4^.

Both chest x-rays^5,6^ and chest computed tomography (CT) have helped guide diagnosis and treatment of COVID-19^7,8^, but there is a paucity of studies describing the association between COVID-19 and physical characteristics of the conducting airways. Previous studies provide evidence of an increase in diameter of the trachea proportional to severity of COVID-19 pneumonia^9^, suggesting that severe inflammation is associated with edema in the trachea and an increase in diameter of the trachea among patients with COVID-19^10^. However, previous studies are limited by focusing on luminal area of the trachea or by not including a comparator group without COVID-19^9,10^.

Accordingly, the primary objective of our study was to determine the relationship between central conducting airway diameter and COVID-19. This retrospective, case–control study used chest CT scans to test the hypothesis that people with COVID-19 would have larger central conducting airways than healthy controls. Additionally, because there are sex-related differences in airway size across the lifespan^11,12^, we included similar data for males and females to assess potential sex-related interactions in the hypothesized airway changes associated with COVID-19.

## Methods

### Ethical approval

This retrospective study was approved by the Institutional Review Board at the Mayo Clinic (IRB no. 17-008537) and conformed to the standards of the Declaration of Helsinki, except registration in a database. Images were collected as part of routine clinical care. Informed consent was waived as no identifiers were used, the data already existed, the research did not affect patient care and the patients’ parent/legal guardian did not opt out of their data being used for research. This consent waiver was approved by the Mayo Clinic Institutional Review Board.

### Patients

Using three-dimensional reconstruction from low-dose high-resolution CT, we retrospectively assessed airway luminal cross-sectional area in patients with COVID-19 and in healthy, sex- and height-matched controls. The CT scans for people with COVID-19 were collected between March 2020 and August 2021. The healthy control cohort represents a historical reference group and CT scans for were collected before the COVID-19 pandemic between March 2009 and March 2018. Notably, because there were demographic and clinical differences in patients who tested positive for COVID-19 during different periods of SARS-CoV-2 variant predominance^13,14^, we did not include data from the Delta “wave” (B.1.617.2) and subsequent “waves” of SARS-CoV-2 variant predominance.

**Figure 1:**
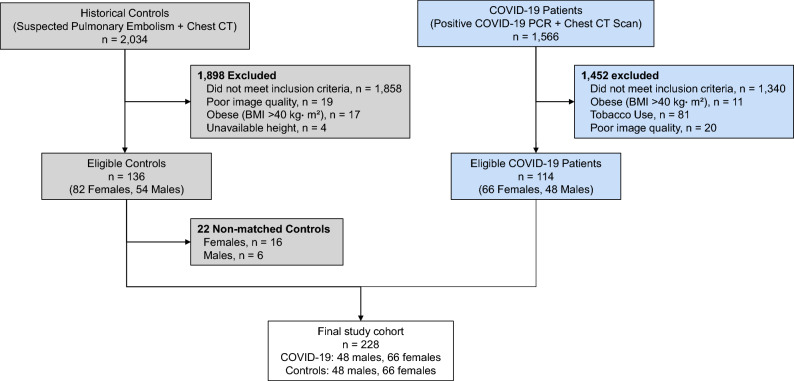
Flow chart of patient eligibility and control matching for the study.

The subject inclusion paradigm is displayed in Fig. 1. For both cohorts (patients with COVID-19 and controls), only adult patients (greater than 17 years of age) were included. For the COVID-19 cohort, patients who were diagnosed with COVID-19 infection (confirmed via polymerase chain reaction-based testing) and who underwent chest CT after COVID-19 diagnosis were considered for inclusion. Exclusion criteria was similar for both groups, and included: heart failure, history of congenital heart/lung disease, rheumatologic disorders (e.g. systemic lupus erythematosus, limited scleroderma, systemic sclerosis, sarcoidosis, or vasculitis), respiratory conditions (e.g. interstitial lung disease, chronic obstructive pulmonary disease, asthma, cystic fibrosis, history of pulmonary embolism, recent or ongoing infection, pulmonary nodules, or pulmonary malignancy), pleural effusion, obstructive sleep apnea, end-stage kidney disease on dialysis, liver disease, ascites, history of pulmonary hypertension, any surgical intervention to the lungs, any tobacco use, and body mass index greater than 40 kg m^−2^.

For the COVID-19 cohort, 1566 patients met the initial inclusion criteria, and their medical history was screened for the pre-specified exclusion criteria. After exclusions, 134 patients with COVID-19 met the criteria, and their images were analyzed for airway luminal area. During the analysis, 20 additional patients with COVID-19 (*n* = 12 males, 8 females) were excluded due to poor quality images—defined by the assessor being unable to visualize all necessary airways. The final cohort of patients with COVID-19 consisted of 114 patients, including 48 males and 66 females.

For the control cohort, 136 patients (54 males, 82 females) who were included in our previous study were considered for inclusion^11^. Potential control patients were individually, one-to-one matched. The cohorts were stratified by sex, and a nearest neighbor matching algorithm was used to match patients based on height (Fig. S1). The final cohort of control patients without COVID-19 consisted of 114 patients, including 48 males and 66 females.

### Image acquisition

The technical specifications associated with image acquisition have been previously described^11^, and are briefly described herein. A posterior-anterior and lateral topogram is obtained at 120 kV and 35 mA. Spiral acquisitions with a pitch of 1.2 are utilized. Kilovoltage is set at 120 with a standard milliampere-second value of 140. Post imaging reconstructions are obtained in the axial and coronal plane using a B46 kernel. Slice thicknesses of 1.5 mm and 3 mm are reconstructed. Maximal intensity projections in the axial and coronal planes are completed with a slice thickness of 10 mm and reconstruction increment of 2.5 mm. Our institution and this project used standardized CT algorithms. Images were acquired at end-inspiration after patients were requested to take a large inspiration and hold their breath. Notably, patients were not instructed to maximally inhale to total lung capacity, thus, lung volumes were not able to be matched between patients (see “Limitations” below). Lung volume was determined during image analysis and was expressed as a percent of predicted total lung capacity based on the subjects’ demographics.

### Data analysis

As previously described^11^, images were analyzed using commercially available software (TeraRecon, AQI, Foster City, CA, USA). The software algorithm isolates the airways from other tissue and creates a three-dimensional reconstruction. The cross-sectional area of the conducting airways was measured at three points (corresponding to the proximal, middle, and distal point of each airway) for each of the following airways: the trachea, right and left main bronchus, left and right upper lobes, intermediate bronchus, and left lower lobe. Anatomical bifurcations defined the proximal and distal point of the measured airways. Additionally, lengths of the trachea, right and left main bronchus, and intermediate bronchus were assessed.

### Statistical analysis

Descriptive statistics are presented as mean ± standard deviation (SD) within the text and tables. Separate univariate analyses of variance were used to compare metrics of central airway size between patients previously diagnosed with COVID-19 and height- and sex-matched controls. Statistical models were performed in duplicate using two representations of luminal airway size— the measurement at the middle of the airway and the average of three measurements (proximal, middle, and distal points). Interpretation of findings did not differ between the statistical models, and findings using the average of three measurements of the airway are presented in the text. Findings using the measurement at the middle of the airway are presented in supporting information (Tables S1 and S2).

Given the known modifying effects of sex, analytical models were performed in duplicate with and without dichotomized models by sex. We also performed exploratory analyses on two subgroups of patients with COVID-19 dichotomized based on hospitalization status using univariate analyses of variance. Categorical variables (group, sex, hospitalization status) and patient characteristics (age, height, weight, and body mass index) were used to construct a decision tree based on the exhaustive Chi-Square Automatic Interaction Detection (CHAID) algorithm^15^ to predict luminal size of each airway. CHAID analysis builds a predictive model to determine the best cutoffs for the input variables to predict an outcome. CHAID creates all possible cross-tabulations for each categorical predictor until the best outcome is achieved and no further splitting can be performed.

Assumptions of normality were confirmed with Shapiro–Wilk tests and assumptions of homoscedasticity were confirmed with Levene’s test. Reported p-values are two-sided, and the interpretation of findings was based on *p* < 0.05. Analyses were performed using IBM Statistical Package for Social Sciences (version 28, Armonk, New York, USA). Figures were created using GraphPad Prism software (version 9, La Jolla, California, USA).

## Results

### Patient characteristics

Patient characteristics are presented in Table 1, stratified by group and sex. Patients with COVID-19 were matched according to sex and height to a control cohort. Control patients were heavier, had larger body mass index, and among females were older compared to patients with COVID-19. For males, there were no differences in absolute lung volume or % predicted lung volume at which the images were obtained between patients with COVID-19 and controls. In contrast, females with COVID-19 had larger absolute lung volume and % predicted lung volume at which the images were obtained compared to controls (*p* < 0.001).

**Table 1:** Patient demographics.

Variable	Males			Females			Sex Diff
	COVID-19	Control	*p*-value	COVID-19	Control	*p*-value	*p*-value
Cohort size, *n*	48	48	–	66	66	–	–
Age, years	57.4 ± 18.2	52.5 ± 18.4	0.196	56.9 ± 16.6	49.7 ± 17.6	**0.017**	0.498
Height, cm	179 ± 7	180 ± 7	0.558	163 ± 7	164 ± 6	0.507	** < 0.001**
Weight, kg	88.0 ± 15.9	95.7 ± 14.5	**0.015**	72.3 ± 13.8	81.2 ± 17.0	**0.001**	** < 0.001**
BMI, kg m^−2^	27.5 ± 4.4	29.6 ± 4.5	**0.022**	27.2 ± 5.1	30.2 ± 5.8	**0.002**	0.817
Hospitalization, *n* (%)	22 (46%)	–	–	15 (23%)	–	–	–

Data are reported as count or mean ± standard deviation (SD). Data are compared using one-way analysis of variance. *p*-values are reported for between group comparisons (COVID-19 vs. control) for males and females separately, and between sex comparisons (males vs. females) for both groups pooled. BMI, body mass index.

Significant values are in bold.

### Association of airway size and COVID-19

**Figure 2:**
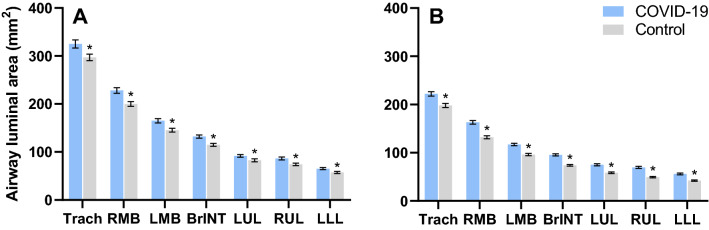
The luminal area of the conducting airways in the height-matched males (**A**) and females (**B**). Data are presented as mean ± SE. Trach, Trachea; RMB, right main bronchus; RUL; right upper lobe; BrINT; bronchus intermediate; LMB, left main bronchus; LUL, left upper lobe; LLL, left lower lobe.

For all central conducting airways, luminal area was ~ 13% larger among males and females with COVID-19 compared to sex- and height-matched controls, Fig. 2. A decision tree model based on the most significant data-splitting factors with the exhaustive CHAID method had an overall classification accuracy of ~ 30% (*n* = 232). Sex and group (COVID-19 vs. control) were included in each decision tree, and four terminal nodes were employed—typified by a 2 × 2 contingency table. These data suggest that both sex and COVID-19 are predictors of airway luminal area.

### Sex-related differences

As expected, males were taller, weighed more, and had greater lung volumes than females (all *p* < 0.001; Tables 1 and 2). In agreement with our previous findings^11^, airway luminal area was ~ 30% greater in males than females (Table 2). However, there were no sex-related interactions in any measurements of luminal airway size, suggesting that the association between COVID-19 and luminal airway size is not different between males and females.

**Table 2:** Airway size of males and females previously diagnosed with COVID-19 and a height- and age-matched control cohort.

Airway luminal size	Males			Females			Sex Diff
	COVID-19	Control	*p*-value	COVID-19	Control	*p*-value	*p*-value
Lung volume, mL	4472 ± 1590	4167 ± 1608	0.355	3785 ± 1035	2879 ± 758	** < 0.001**	** < 0.001**
Pred of scan TLC, %	61.5 ± 19.4	57.1 ± 20.5	0.284	75.6 ± 17.3	57.3 ± 14.4	** < 0.001**	**0.005**
Trachea, mm^2^	325 ± 58	297 ± 48	**0.012**	222 ± 38	198 ± 33	** < 0.001**	** < 0.001**
Right main bronchus, mm^2^	228 ± 41	200 ± 33	** < 0.001**	163 ± 31	132 ± 26	** < 0.001**	** < 0.001**
Right upper lobe, mm^2^	86.6 ± 20.7	74.1 ± 18.8	**0.003**	69.6 ± 16.8	49.4 ± 12.2	** < 0.001**	** < 0.001**
Bronchus Intermediate, mm^2^	132.1 ± 23.3	114.9 ± 20.7	** < 0.001**	95.6 ± 17.0	73.9 ± 14.3	** < 0.001**	** < 0.001**
Left main bronchus, mm^2^	165.0 ± 30.8	145.4 ± 27.6	**0.001**	117.0 ± 21.2	96.1 ± 18.5	** < 0.001**	** < 0.001**
Left upper lobe, mm^2^	91.8 ± 18.2	82.7 ± 21.9	**0.029**	75.0 ± 16.4	58.5 ± 14.4	** < 0.001**	** < 0.001**
Left lower lobe, mm^2^	65.3 ± 14.1	57.4 ± 14.9	**0.009**	56.0 ± 14.2	42.1 ± 10.5	** < 0.001**	** < 0.001**

Data are reported as mean ± standard deviation (SD). Data are compared using one-way analysis of variance. *p*-values are reported for between group comparisons (COVID-19 vs. control) for males and females separately, and between sex comparisons (males vs. females) for both groups pooled. Pred of scan TLC, predictive relation lung volume based on the computed tomography scan, absolute measured volume, and predicted total lung capacity.

Significant values are in bold.

### Exploratory analyses based on hospitalization status

Although the primary objective was to determine the association between COVID-19 and luminal airway size, additional exploratory analyses were performed among COVID-19 patients based on hospitalization status. Twenty-two of 48 (46%) males with COVID-19 and 15 of 68 (22%) females with COVID-19 required COVID-19 related hospitalization (Table 1). Compared to patients who did not require COVID-19-related hospitalization, patients who were hospitalized with COVID-19 were not different in age, height, weight, or body mass index (Table 3), with the exception of larger body mass index among females (*p* = 0.023). For both males and females, patients who were hospitalized with COVID-19 had greater lung volume and % predicted lung volume at which the images were obtained compared to patients who did not require COVID-19-related hospitalization. For the preponderance of airways, there were no differences in luminal size between patients COVID-19 stratified by hospitalization status, except for the left upper lobe for males and females and the right upper lobe for males.

**Table 3:** Airway size of males and females previously diagnosed with COVID-19 comparing subsets of patients that were hospitalized and patients that were not hospitalized for COVID-19.

Variable	Males			Females		
	Hospitalized	Not hospitalized	*p*-value	Hospitalized	Not hospitalized	*p*-value
Cohort size, n	22	26	–	15	51	–
Age, years	62.0 ± 14.8	53.5 ± 20.1	0.108	60.0 ± 15.4	56.0 ± 17.0	0.420
Height, cm	178 ± 6	180 ± 9	0.345	161 ± 7	164 ± 7	0.178
Weight, kg	87.3 ± 13.4	88.5 ± 18.0	0.792	77.6 ± 13.3	70.7 ± 13.7	0.088
BMI, kg m^−2^	27.5 ± 3.4	27.5 ± 5.1	0.971	29.9 ± 4.3	26.4 ± 5.0	**0.016**
Lung volume, mL	3765 ± 1416	5070 ± 1502	**0.003**	3164 ± 864	3967 ± 1017	**0.007**
Pred of scan TLC, %	52.6 ± 18.6	69.0 ± 16.9	**0.002**	65.5 ± 17.0	78.6 ± 16.5	**0.009**
Trachea, mm^2^	329 ± 61	322 ± 56	0.675	223 ± 43	222 ± 36	0.963
Right main bronchus, mm^2^	236 ± 49	222 ± 34	0.274	170 ± 43	162 ± 27	0.375
Right upper lobe, mm^2^	92.9 ± 23.4	81.3 ± 16.9	0.054	69.6 ± 17.4	69.6 ± 16.8	0.993
Bronchus intermediate, mm^2^	133.7 ± 23.3	130.8 ± 23.6	0.674	97.5 ± 20.7	95.1 ± 15.9	0.630
Left main bronchus, mm^2^	166.9 ± 35.8	163.5 ± 26.6	0.707	117.9 ± 29.6	116.8 ± 18.4	0.860
Left upper lobe, mm^2^	85.7 ± 18.0	97.0 ± 17.1	**0.031**	66.9 ± 13.4	77.4 ± 16.6	**0.029**
Left lower lobe, mm^2^	65.5 ± 13.7	65.1 ± 14.8	0.920	55.1 ± 14.4	56.3 ± 14.3	0.778

Data are reported as mean ± standard deviation (SD). Data are compared using one-way analysis of variance. *p*-values are reported for between group comparisons (hospitalized vs. not hospitalized) for males and females separately. BMI, body mass index; Pred of scan TLC, predictive relation lung volume based on the computed tomography scan, absolute measured volume, and predicted total lung capacity.

Significant values are in bold.

## Discussion

### Principal findings

The primary aim of this study was to evaluate central conducting airway size in people previously diagnosed with COVID-19 in comparison to healthy, sex- and height-matched adults. Consistent with our hypothesis, we found that both males and females previously diagnosed with COVID had larger luminal areas of central conducting airways compared to matched controls, including trachea, left and right main bronchus, intermediate bronchus, left and right upper lobe, and left lower lobe. Additional exploratory analyses also demonstrated that compared to patients who did not require COVID-19-related hospitalization, patients who were hospitalized with COVID-19 had no differences in luminal areas of central conducting airways. These findings suggest that symptomatic COVID-19 infection may be associated with pathologically larger central conducting airway luminal areas than healthy, sex- and height-matched controls.

### Pathophysiology of COVID-19 in the conducting airways

Disease processes can impart differences on the luminal area of the conducting airways, which transport gases without participating in gas exchange^11,16–19^. In the context of COVID-19, SARS-CoV-2 enters human bodies through respiratory droplets and attaches to epithelial cells of the conducting airways, suppressing the mucociliary apparatus that removes secretions and inhaled particles^2,3,20,21^. The resulting accumulation of contaminated mucous and airway edema may contribute to the increased luminal areas we observed on CT images of patients with COVID-19^22–24^. The disease course of COVID-19 in the conducting airways has yet to be elucidated. Previous studies demonstrate that airway luminal area may increase proportionate to disease severity and improve with resolution of acute infection^10,25^. Our study supports the finding of larger airways among patients with COVID-19 compared to controls, however, our study does not provide information on chronic changes to the airway following recovery from COVID-19. Airway luminal area is the major determinant of airway resistance and is particularly important when considering the implications of airway resistance in health and disease^26–28^. Although an increase in airway luminal area will decrease the resistance to airflow, this increase in airway luminal area will also contribute to reduced flow for a given pressure. Thus, the increase in airway luminal area can reduce the expectorant role of the conducting airways to clear mucous and debris before reaching the lung. Greater airway luminal area may also contribute to greater deposition of inhaled particles in lower portions of the airway^29^. Paradoxically, then, larger airway size may both predispose and be the result of inflammatory processes which ultimately result in mucous plugging and decreased airflow^30^.

### Sex differences in airway anatomy

In addition to pathologic differences in the luminal area of conducting airways, anatomic differences between males and females are well established in the literature^11,12,31,32^. Males have larger luminal areas than females in the central conducting airways; this difference in airway size may affect resistance to air flow and aerosol deposition across the lifespan^33^. Consistent with previous studies, males in our cohort had larger luminal areas than females in all seven of the central conducting airways that were measured. However, there were no sex-related interactions in the differences observed in the luminal area of conducting airways among people with COVID-19 compared to controls. Thus, sex does not appear to interact with the observed effects of COVID-19 on central conducting airway luminal area.

### Potential clinical implications

Generally, radiographic findings associated with COVID-19 from both chest x-ray^5,6^ and CT reflect a typical lung injury of viral pneumonia^7,8^. The primary radiographic findings are ground-glass opacity and pulmonary consolidation—suggesting the possible presence of organizing pneumonia^8^. Although effective vaccines and therapeutics are available in many countries, about one-third of COVID-19 survivors have residual abnormalities on chest CT 1 year after COVID-19^34^. Thus, familiarity with sequelae of COVID-19 pneumonia on chest imaging may be important to evaluate potential causes of chronic residual abnormalities or respiratory symptoms after COVID-19^35,36^.

In this context, our findings showing larger central conducting airway luminal area among patients with COVID-19 may have clinical implications for post-COVID conditions—also referred to as “long COVID”, “long-haul COVID”, or “post-acute sequelae of COVID-19”^34^. The larger airway luminal area may reflect traction bronchiectasis and could contribute to post-COVID respiratory symptoms^34,37^. Although no consensus currently exists for imaging management of patients with subacute COVID-19, our findings may suggest that enlarged central conducting airway luminal area may be a consideration in pulmonary sequelae among COVID-19 survivors.

## Limitations

Several limitations resulted from the design of this study, which may highlight areas for future investigation. First, the end-inspiratory lung volume was not standardized to total lung capacity. Rather, subjects were instructed to inspire and hold their breath. Notably, there were no observed differences in the relative lung volume between the two cohorts (COVID-19, control). Additionally, lung volume also has less of an influence on more proximal airways (which the current study assessed) compared with more distal airways^38^. Thus, although caution is required while interpreting absolute airway diameters, our primary comparison between cohorts is likely unaffected. Second, we used nonprobability sampling and a simplistic, cross-sectional design. Although our findings and those of others suggest a relationship between COVID-19 and larger airway luminal size^10,39^, the data should not be used to infer a definitive causal relationship or definitive temporal changes associated with COVID-19. Third, assessments of pulmonary function were not available in this cohort, as such, we were not able to determine the potential relationship between larger airway size and pulmonary function. Fourth, assessments of putative factors were not assessed, including, concentration of angiotensin converting enzyme receptors and changes in the pulmonary interstitium.

## Conclusion

Our findings suggest that males and females previously diagnosed with COVID-19 have larger luminal area of conducting airways compared to healthy sex- and height-matched controls. Further, COVID-19-related hospitalization was not associated with changes in luminal area of conducting airways among patients with COVID-19. A key limitation of the study is that the COVID-19 disease course was not characterized.

## Supplementary Information

Supplementary Information.

## Data Availability

Datasets generated during this study may also be available from corresponding authors on reasonable request. Requestors may be required to sign a data use agreement. Data sharing must be compliant with all applicable Mayo Clinic policies.

## References

[1] Lai CC, Shih TP, Ko WC, Tang HJ, Hsueh PR (2020). Severe acute respiratory syndrome coronavirus 2 (SARS-CoV-2) and coronavirus disease-2019 (COVID-19): The epidemic and the challenges. Int. J. Antimicrob. Agents.

[2] Bridges JP, Vladar EK, Huang H, Mason RJ (2022). Respiratory epithelial cell responses to SARS-CoV-2 in COVID-19. Thorax.

[3] Jia HP (2005). ACE2 receptor expression and severe acute respiratory syndrome coronavirus infection depend on differentiation of human airway epithelia. J. Virol..

[4] Brosnahan SB, Jonkman AH, Kugler MC, Munger JS, Kaufman DA (2020). COVID-19 and respiratory system disorders: Current knowledge, future clinical and translational research questions. Arterioscler. Thromb. Vasc. Biol..

[5] Roig-Marin N, Roig-Rico P (2022). Ground-glass opacity on emergency department chest X-ray: A risk factor for in-hospital mortality and organ failure in elderly admitted for COVID-19. Postgrad. Med..

[6] Roig-Marin N, Roig-Rico P (2022). The deadliest lung lobe in COVID-19: A retrospective cohort study of elderly patients hospitalized for COVID-19. Postgrad. Med..

[7] Aljondi R, Alghamdi S (2020). Diagnostic value of imaging modalities for COVID-19: Scoping review. J. Med. Internet Res..

[8] Wang Y (2020). Temporal changes of CT findings in 90 patients with COVID-19 pneumonia: A longitudinal study. Radiology.

[9] Unlu S, Ilgar M, Akcicek M (2021). The evaluation of the trachea as a new parameter in determining the prognosis of COVID-19: First pilot study. Eur. Rev. Med. Pharmacol. Sci..

[10] Sun Z (2020). Computed tomography evaluation of airway changes in adult patients with COVID-19 pneumonia. J. Coll. Physicians Surg. Pak..

[11] Dominelli PB (2018). Sex differences in large conducting airway anatomy. J. Appl. Physiol..

[12] Ripoll JG (2020). Sex differences in paediatric airway anatomy. Exp. Physiol..

[13] Fisman DN, Tuite AR (2021). Evaluation of the relative virulence of novel SARS-CoV-2 variants: A retrospective cohort study in Ontario, Canada. CMAJ.

[14] Christensen PA (2022). Signals of significantly increased vaccine breakthrough, decreased hospitalization rates, and less severe disease in patients with coronavirus disease 2019 caused by the omicron variant of severe acute respiratory syndrome coronavirus 2 in Houston, Texas. Am. J. Pathol..

[15] Kass GV (1980). An exploratory technique for investigating large quantities of categorical data. J. R. Stat. Soc. Ser. C Appl. Stat..

[16] Sakai H (2010). Age-related changes in the trachea in healthy adults. Adv. Exp. Med. Biol..

[17] Griscom NT, Wohl ME (1985). Dimensions of the growing trachea related to body height. Length, anteroposterior and transverse diameters, cross-sectional area, and volume in subjects younger than 20 years of age. Am. Rev. Respir. Dis..

[18] D'Anza B, Knight J, Greene JS (2015). Does body mass index predict tracheal airway size?. Laryngoscope.

[19] Hedenstierna G, Sandhagen B (2006). Assessing dead space. A meaningful variable?. Minerva. Anestesiol..

[20] Ren X (2006). Analysis of ACE2 in polarized epithelial cells: Surface expression and function as receptor for severe acute respiratory syndrome-associated coronavirus. J. Gen. Virol..

[21] To KF, Lo AW (2004). Exploring the pathogenesis of severe acute respiratory syndrome (SARS): The tissue distribution of the coronavirus (SARS-CoV) and its putative receptor, angiotensin-converting enzyme 2 (ACE2). J. Pathol..

[22] Salehi S, Abedi A, Balakrishnan S, Gholamrezanezhad A (2020). Coronavirus disease 2019 (COVID-19): A systematic review of imaging findings in 919 patients. AJR Am. J. Roentgenol..

[23] Zhou S, Wang Y, Zhu T, Xia L (2020). CT features of coronavirus disease 2019 (COVID-19) pneumonia in 62 patients in Wuhan, China. AJR Am. J. Roentgenol..

[24] Li Y, Xia L (2020). Coronavirus disease 2019 (COVID-19): Role of chest CT in diagnosis and management. AJR Am. J. Roentgenol..

[25] Lindahl A (2021). Small airway function in Finnish COVID-19 survivors. Respir. Res..

[26] Barnes PJ (2015). Chronic obstructive pulmonary disease. Nat. Rev. Dis. Primers.

[27] West JB (1975). Respiratory Physiology: The Essentials.

[28] Lumb AB, Nunn JF (2010). Nunn's Applied Respiratory Physiology.

[29] Christou S (2021). Anatomical variability in the upper tracheobronchial tree: Sex-based differences and implications for personalized inhalation therapies. J. Appl. Physiol..

[30] Fahy JV, Dickey BF (2010). Airway mucus function and dysfunction. N. Engl. J. Med..

[31] Sheel AW (2009). Evidence for dysanapsis using computed tomographic imaging of the airways in older ex-smokers. J. Appl. Physiol. (1985).

[32] Peters CM (2021). Airway luminal area and the resistive work of breathing during exercise in healthy young females and males. J. Appl. Physiol..

[33] Mann LM, Angus SA, Doherty CJ, Dominelli PB (2021). Evaluation of sex-based differences in airway size and the physiological implications. Eur. J. Appl. Physiol..

[34] Kanne JP, Little BP, Schulte JJ, Haramati A, Haramati LB (2022). Long-term lung abnormalities associated with COVID-19 pneumonia. Radiology.

[35] Vadasz I (2021). Severe organising pneumonia following COVID-19. Thorax.

[36] Ng BH, Ban AY, Nik Abeed NN, Faisal M (2021). Organising pneumonia manifesting as a late-phase complication of COVID-19. BMJ Case Rep..

[37] Watanabe A (2022). One-year follow-up CT findings in COVID-19 patients: A systematic review and meta-analysis. Respirology.

[38] Kambara K (2014). Effect of lung volume on airway luminal area assessed by computed tomography in chronic obstructive pulmonary disease. PLoS ONE.

[39] Xia J (2020). Increased physiological dead space in mechanically ventilated COVID-19 patients recovering from severe acute respiratory distress syndrome: A case report. BMC Infect. Dis..

