# Supplementary material for: Greater central airway luminal area in people with COVID-19: a case–control series
Source: Sci Rep. 2022 Oct 26;12:17970. doi: 10.1038/s41598-022-22005-6 (PMC9606286; doi:10.1038/s41598-022-22005-6)
Supplement: Supplementary file 1 — Supplementary Information. [file 41598_2022_22005_MOESM1_ESM.docx]

Supporting Information for

*Greater Central Airway Luminal Area in People with COVID-19:
A Case-Control Series*

**Jeffrey L. Jeltema^1,2^ | Ellen K. Gorman^2^ | Erik A. Ovrom^1,2^ | Jonathon W. Senefeld^2^ |**
**Juan G. Ripoll^2^ | Paolo B. Dominelli^3^ | Michael J. Joyner^2^ | Brian T. Welch^4^ |
 Chad C. Wiggins^2^**

**Affiliations**

1 | Alix School of Medicine, Mayo Clinic, Rochester, Minnesota

2 | Department of Anesthesiology and Perioperative Medicine, Mayo Clinic, Rochester, Minnesota

3 | Department of Kinesiology, University of Waterloo, Waterloo, Ontario, Canada

4 | Department of Radiology, Mayo Clinic, Rochester, Minnesota

*This appendix has been provided by the authors to give readers additional information about their work.*

**Greater Central Airway Luminal Area in People with COVID-19:
A Case-Control Series**

**Supplementary Appendix**

**Contents**

**Supplementary Tables**

**Table S1.** Airway size of males and females previously diagnosed with COVID-19 and a height- and age-matched control cohort. **3**

**Table S2.** Airway size of males and females previously diagnosed with COVID-19 comparing subsets of patients that were hospitalized and patients that were not hospitalized for COVID-19. **4**

**Supplementary Figures**

**Figure S1.** Matches chosen using 1:1 nearest neighbor matching algorithm based on height of males (blue circles) and females (red triangles) with COVID-19 and controls **5**

**Supplementary Tables**

| **Airway Luminal Size** | **Males** | | |  | **Females** | | |  | **Sex Diff.** |
| --- | --- | --- | --- | --- | --- | --- | --- | --- | --- |
|  | **COVID-19** | **Control** | ***p*-value** |  | **COVID-19** | **Control** | ***p*-value** |  | ***p*-value** |
| Trachea, mm^2^ | 327 ± 89 | 291 ± 58 | **0.019** |  | 218 ± 38 | 198 ± 38 | **0.003** |  | **<0.001** |
| Right main bronchus, mm^2^ | 225 ± 43 | 193 ± 33 | **<0.001** |  | 162 ± 30 | 130 ± 26 | **<0.001** |  | **<0.001** |
| Right upper lobe, mm^2^ | 80.9 ± 22.4 | 71.9 ± 19.6 | **0.040** |  | 65.7 ± 17.6 | 48.5 ± 12.7 | **<0.001** |  | **<0.001** |
| Bronchus Intermediate, mm^2^ | 127.5 ± 24.1 | 109.4 ± 21.8 | **<0.001** |  | 91.2 ± 16.3 | 68.0 ± 16.9 | **<0.001** |  | **<0.001** |
| Left main bronchus, mm^2^ | 148.4 ± 31.9 | 127.4 ± 27.9 | **<0.001** |  | 107.3 ± 23.0 | 81.5 ± 18.1 | **<0.001** |  | **<0.001** |
| Left upper lobe, mm^2^ | 91.1 ± 19.2 | 82.4 ± 22.7 | **0.044** |  | 74.4 ± 16.7 | 57.4 ± 14.1 | **<0.001** |  | **<0.001** |
| Left lower lobe, mm^2^ | 58.1 ± 15.3 | 55.9 ± 16.0 | 0.503 |  | 49.1 ± 13.0 | 41.4 ± 11.3 | **<0.001** |  | **<0.001** |

**Table S1.** Airway size of males and females previously diagnosed with COVID-19 and a height- and age-matched control cohort. Data are reported as mean ± standard deviation (SD) and represent measurements at the middle of the airway. Data are compared using one-way analysis of variance. *p*-values are reported for between group comparisons (COVID-19 vs. control) for males and females separately, and between sex comparisons (males vs. females) for both groups pooled.

| **Variable** | **Males** | | |  | **Females** | | |
| --- | --- | --- | --- | --- | --- | --- | --- |
|  | **Hospitalized** | **Not Hospitalized** | ***p*-value** |  | **Hospitalized** | **Not Hospitalized** | ***p*-value** |
| Trachea, mm^2^ | 336 ± 94 | 320 ± 84 | 0.515 |  | 223 ± 44 | 217 ± 36 | 0.571 |
| Right main bronchus, mm^2^ | 232 ± 51 | 219 ± 35 | 0.304 |  | 168 ± 38 | 160 ± 27 | 0.392 |
| Right upper lobe, mm^2^ | 89.3 ± 26.7 | 73.8 ± 15.2 | **0.015** |  | 66.4 ± 17.5 | 65.5 ± 17.8 | 0.859 |
| Bronchus Intermediate, mm^2^ | 129.3 ± 24.6 | 126.0 ± 24.0 | 0.638 |  | 94.3 ± 19.4 | 90.4 ± 15.4 | 0.419 |
| Left main bronchus, mm^2^ | 152.2 ± 36.0 | 145.2 ± 28.3 | 0.458 |  | 106.7 ± 28.2 | 107.5 ± 21.6 | 0.906 |
| Left upper lobe, mm^2^ | 84.2 ± 19.2 | 97.0 ± 17.4 | **0.020** |  | 66.3 ± 13.5 | 76.8 ± 16.9 | **0.030** |
| Left lower lobe, mm^2^ | 59.1 ± 14.3 | 57.3 ± 16.4 | 0.693 |  | 47.4 ± 13.3 | 49.6 ± 13.0 | 0.562 |

**Table S2.** Airway size of males and females previously diagnosed with COVID-19 comparing subsets of patients that were hospitalized and patients that were not hospitalized for COVID-19. Data are reported as mean ± standard deviation (SD) and represent measurements at the middle of the airway. Data are compared using one-way analysis of variance. *p*-values are reported for between group comparisons (hospitalized vs. not hospitalized) for males and females separately.

**Supplementary Figures**

**

**

**Figure S1.** Matches chosen using 1:1 nearest neighbor matching algorithm based on height of males (blue circles) and females (red triangles) with COVID-19 and controls. Symbols represent height of patients with COVID-19 and controls, and lines represent the matched pair.
